# Retinoic Acid Induces an IFN-Driven Inflammatory Tumour Microenvironment, Sensitizing to Immune Checkpoint Therapy

**DOI:** 10.3389/fonc.2022.849793

**Published:** 2022-03-24

**Authors:** Caitlin M. Tilsed, Thomas H. Casey, Emma de Jong, Anthony Bosco, Rachael M. Zemek, Joanne Salmons, Graeme Wan, Michael J. Millward, Anna K. Nowak, Richard A. Lake, Willem Joost Lesterhuis

**Affiliations:** ^1^ School of Biomedical Sciences, University of Western Australia, Crawley, WA, Australia; ^2^ National Centre for Asbestos Related Diseases, Institute for Respiratory Health, Nedlands, WA, Australia; ^3^ Telethon Kids Institute, University of Western Australia, Nedlands, WA, Australia; ^4^ Medical School, University of Western Australia, Crawley, WA, Australia; ^5^ Department of Medical Oncology, Sir Charles Gairdner Hospital, Nedlands, WA, Australia

**Keywords:** retinoic acid, immune checkpoint therapy, cancer, interferon, mesothelioma

## Abstract

With immune checkpoint therapy (ICT) having reshaped the treatment of many cancers, the next frontier is to identify and develop novel combination therapies to improve efficacy. Previously, we and others identified beneficial immunological effects of the vitamin A derivative tretinoin on anti-tumour immunity. Although it is known that tretinoin preferentially depletes myeloid derived suppressor cells in blood, little is known about the effects of tretinoin on the tumour microenvironment, hampering the rational design of clinical trials using tretinoin in combination with ICT. Here, we aimed to identify how tretinoin changed the tumour microenvironment in mouse tumour models, using flow cytometry and RNAseq, and we sought to use that information to establish optimal dosing and scheduling of tretinoin in combination with several ICT antibodies in multiple cancer models. We found that tretinoin rapidly induced an interferon dominated inflammatory tumour microenvironment, characterised by increased CD8+ T cell infiltration. This phenotype completely overlapped with the phenotype that was induced by ICT itself, and we confirmed that the combination further amplified this inflammatory milieu. The addition of tretinoin significantly improved the efficacy of anti-CTLA4/anti-PD-L1 combination therapy, and staggered scheduling was more efficacious than concomitant scheduling, in a dose-dependent manner. The positive effects of tretinoin could be extended to ICT antibodies targeting OX40, GITR and CTLA4 monotherapy in multiple cancer models. These data show that tretinoin induces an interferon driven, CD8+ T cell tumour microenvironment that is responsive to ICT.

## Introduction

In the last decade, cancer immunotherapy has resulted in improved outcomes in some cancers, such as melanoma and non-small lung cancer (1). In particular, treatment with antibodies that target immune checkpoints programmed death receptor-1 (PD-1), its ligand PDL1, or cytotoxic T lymphocyte associated protein 4 (CTLA-4) have shown durable complete regressions in a substantial percentage of patients, resulting in FDA approval in an expanding number of indications (2). However, despite these positive results in a portion of patients with certain cancers, still many patients do not derive a therapeutic benefit, and some cancers are more resistant than others (1). For example, sarcoma, mesothelioma, pancreatic cancer and microsatellite-stable colorectal cancers have low response rates to antibodies targeting PD1, PD-L1 or CTLA-4 (3–5).

Due to the low or modest response rates in many cancers, there is an urgent need to develop new effective combinations. Given that currently over 2000 clinical trials are actively recruiting patients with any combination of immune checkpoint therapy (ICT) with another drug, this development would ideally be based on solid preclinical evidence, as to accelerate identification of candidate combinations and minimize patient exposure to futile treatments with a risk of sometimes severe side effects (6). In addition, given the long clinical development pipeline of novel compounds, using existing, FDA-approved drugs in combination with checkpoint inhibitors is a highly attractive avenue (7).

We developed bilateral tumour mouse models that allowed us to identify mechanisms operating in the tumour during treatment with ICTs in responding and non-responding mice, within the same tumour model (8, 9). Using these models, we can map gene expression signatures associated with response and identify repurposed drugs that can induce this response in combination with ICT. Previously, we identified that combination therapy of retinoic acid and anti-CTLA4 antibody was highly efficacious in a mesothelioma mouse model (10). Tretinoin is a vitamin A derivative that has both direct anti-cancer effects (11, 12) and immunomodulatory properties (13). These include depleting myeloid derived suppressor cells (MDSCs) (14, 15), inducing expansion and activation of CD8+ T cells (16, 17) and promoting CD4 effector responses (18).

Here, we aimed to understand the effects of tretinoin treatment on the tumor microenvironment and to determine the optimal dose and schedule of tretinoin in combination with multiple ICT antibodies in multiple cancer models to facilitate further clinical translation.

## Materials and Methods

### Cells

The AB1-HA murine mesothelioma, RENCA renal cell carcinoma and WEHI-164 fibrosarcoma cell line was obtained from Cell Bank Australia. Cell lines were subject to regular mycoplasma testing and PCR verification and validated by CellBank Australia. Cells were maintained in RPMI 1640 (Invitrogen) supplemented with 20 mM Hepes, 0.05 mM 2-mercaptoethanol, penicillin (100 U/ml; CSL), gentamicin (50 mg/ml; David Bull Labs), and 10% newborn calf serum (NCS; Thermo Fisher Scientific). Media used for AB1HA cell culture was further supplemented with 50 mg/ml Geneticin (G418; Life Technologies, Mulgrave, VIC, Australia). Cells were passaged upon reaching 80-85% confluence and passaged 4-5 times prior to inoculation in mice.

### Mouse Tumour Cell Inoculation and Monitoring

Female and male BALB/c mice (8-10 weeks) were obtained from the Animal Resource Centre (Murdoch, WA, Australia) and housed at the Harry Perkins Bio Resources Facility (Nedlands, WA, Australia). For subcutaneous inoculation, adherent cells were removed from the flask using trypsin and washed three times with PBS. Cells were counted using a hemocytometer with viability determined by trypan blue exclusion with a viability of at least 90% to be inoculated. 5x10^5^ cells were inoculated in one or both flanks of mice in 100 µl of PBS. Mice were randomised to treatment groups and researchers measuring tumour size (the primary experimental endpoint) were blinded for treatment allocation. Tumour growth was measured with standard mechanical callipers, 3 times a week until tumours reached 70mm^2^ by which they were then monitored daily. Mice were culled when the tumour exceeded 95mm^2^ in size. All experiments were conducted in compliance with the institutional guidelines provided by the Harry Perkins Institute for Medical Research animal ethics committee (approval numbers 091 and 156).

### Hematoxylin and Eosin Staining

After removal, tumor samples were fixed with 4% paraformaldehyde for 48 - 72 h and embedded in paraffin. 5 µm sections were cut by microtome (Thermo Fisher Scientific), placed on slides and deparaffinised using the following protocol; 2 rounds of xylene (3 min each), 2 rounds of 100% ethanol (2 min each), 95% ethanol (1 min), 70% ethanol (1 min), 40% ethanol (1 min), 3 rounds of distilled H2O (3 min each). Slides were stained with Mayers hematoxylin (Sigma-Aldrich) for 10 min and rinsed with running tap water, the counterstain was performed with acidified Eosin Y solution (Sigma-Aldrich) (0.5% glacial acetic acid) for 45 s. The dehydration protocol is as follows; 40% ethanol (30 s), 70% ethanol (30 s), 95% ethanol (30 s), 2 rounds of 100% ethanol (1 min each), 2 rounds of xylene (3 min each). Mounting was performed with Pertex mounting medium (Histolab), and sections were imaged under a light microscope.

### Treatments

Tretinoin (all-trans retinoic acid ≥98%, HPLC, powder) was purchased from Sigma-Aldrich (now Merck; Macquarie Park, NSW) and suspended in Dimethyl Sulfoxide (DMSO; Sigma-Aldrich) at a concentration of 40 mg/ml for short term storage at -80°C. Mice were treated with a maximum of 10 mg/kg tretinoin for 9 consecutive days, intraperitoneally (i.p.). Tretinoin treatment either commenced on the same day as ICT (concomitant treatment) or commenced 3 days prior to ICT (staggered treatment). PBS was used as a control for ICT and PBS containing 10% DMSO was used as a control for tretinoin treatment. For experiments in RENCA, 10 mg/kg tretinoin was administered *via* oral gavage in 5% DMSO in soybean oil for five consecutive days commencing 5 days prior to ICT (pre-treatment) or on the day of ICT (concomitant). DMSO and soybean oil was used as a vehicle control.

Anti-CTLA-4 (clone: 9H10), anti-PD-L1 (clone: B7-H1), anti-OX40 (clone: OX-86) and anti-GITR (clone: DTA-1) checkpoint blockade *in vivo* monoclonal antibodies were all purchased from BioXcell, West Lebanon, NH. Anti-CTLA-4 (100 µg), anti-OX40 (200 µg), and anti-GITR (100 µg), were given as single i.p doses and anti-PD-L1 (100 µg), was given i.p, 3 times, every 2 days.

For RNAseq and flow cytometry experiments, AB1-HA tumour-bearing BALB/c mice were treated with vehicle, tretinoin, ICT or the combination and tumours harvested. For experiments with response to the ICT/tretinoin combination as a readout, a bilateral tumour model was used (8, 10). In brief, mice were inoculated with two tumours, one on either flank. One tumour was surgically resected after either 5 days of tretinoin, 2 injections of ICT antibody, the combination, or PBS control, with the other tumour remaining *in* situ to provide a readout of treatment efficacy (responder with complete tumour cure or non-responder with tumour outgrowth).

### Tumour Preparation for RNA-seq

For RNA-seq, whole tumours were surgically resected as described above, and the surrounding tissue was removed and immediately submerged in RNAlater (Life Technologies). Samples were stored at 4°C for 24 hours, supernatant was then removed and samples were transferred to −80°C. Frozen tumours were dissociated in TRIzol (Life Technologies) using a TissueRuptor (QIAGEN). RNA was extracted using chloroform and purified on RNeasy MinElute columns (QIAGEN) and RNA integrity was confirmed using a Bioanalyzer (Agilent Technologies). Library preparation and sequencing (100 base pair single-end) were performed by Australian Genome Research Facility, using Illumina NovaSeq standard protocols.

### RNAseq Analysis

Raw sequencing files underwent pre-alignment quality control using FastQC (19) (v0.11.3), followed by alignment to the mouse genome (mm11) using HISAT2 (20) (v2.0.4) and gene-level quantitation using SummariseOverlaps (21). Post-alignment quality assessment was performed with Samstat (22) (v1.5.2). Pairwise comparisons between treatment groups to identify differentially expressed genes were performed with DESeq2 (23), where Benjamini-Hochberg adjusted p-values <0.05and absolute log fold change ≥1 were considered significant. Downstream analysis included upstream regulator analysis (24), gene set enrichment analysis (GSEA) (25), innateDB pathway analysis (26) and CIBERSORT immune cell deconvolution using a 511 mouse gene signature (27) as a reference. Immune populations were grouped into 10 major populations as follows; B cells include memory, naive and plasma cells; CD8^+^ T cells include memory, naive and activated cells; CD4^+^ T cells include memory, naive, follicular, Th1, Th2 and Th17 cells; macrophages include M0, M1 and M2 macrophages; NK cells include activated and resting cells; DCs include activated and immature cells; and the remaining populations are mast cells, Tregs and monocytes. Raw and processed data are accessible through GEO Series accession number GSE186195.

### Flow Cytometry

For flow cytometry, tumours were dissociated into a single cell suspension using the Miltenyi gentleMACs. Spleens were digested with 1 mg/ml type IV collagenase (Worthington Biochemical) and 1 ug/ml DNase (Sigma Aldrich) in RPMI-1640 supplemented with 2% NCS and 20 mM Hepes for 20 mins at RT. 500 µl of 0.1 M EDTA was added and spleens digested for a further 5 minutes to break up DC-T cell rosettes. The cell suspension was underlayed with cold EDTA-DCs and cell recovered by centrifugation at 1600 rpm for 10 mins. Red blood cells were lysed with Pharm Lyse (BD Biosciences) for 5 mins and remaining cells underlayed with EDTA-BSS-FCS. Cells were collected *via* centrifugation and resuspended in EDTA-BSS-FCS for staining. Samples were stained for dead cell exclusion (FVD-ef780 or ZombieUV) for 30 minutes in the dark at room temperature (RT). Antibodies for surface staining were suspended in PBS+2%FCS and incubated for 30 minutes at RT. For intracellular staining, cells were fixed and permeabilized for 10 minutes at RT using the Foxp3/Transcription Factor Staining Buffer Set (eBioscience). Intracellular antibodies were suspended in Permeabilization Buffer (eBiosciences) and incubated for 30 mins at RT. Samples were washed 3x in Permeabilization Buffer, resuspended in Stabilizing Fixative (BD) and stored at 4°C. Data was acquired using the BD LSRFortessa and analysed using FlowJo (BD Biosciences). Flow cytometry panels are detailed in **Supplemental Table 1** and gating strategy is as detailed in **Supplemental Figure 1**.

### Statistical Analysis

For flow cytometry experiments and CIBERSORT data, results are presented as mean ± SD and statistical analysis was performed using Mann-Whitney U tests corrected for multiple comparisons using GraphPad Prism v8. Survival data was analyzed using Log-rank (Mantel-Cox) test in GraphPad Prism v8 and differences in tumour growth was assessed using a mixed model ANOVA (28) with analysis performed in R.

## Results

### Tretinoin Induces an Inflammatory, Interferon-Driven Tumour Microenvironment

Previous studies have demonstrated that the addition of tretinoin to ICT significantly improved response rate and survival, reportedly due to systemic MDSC depletion (29, 30). Additionally, our own preclinical work identified a gene expression signature associated with response to ICT, overlapped with gene signatures derived from cell lines treated with tretinoin using drug repurposing database LINCS (10). However, the effects of tretinoin on the cellular and molecular characteristics of the tumour microenvironment have not been comprehensively determined. We therefore first performed RNA sequencing and flow cytometry on tumour-bearing mice treated with tretinoin.

**Figure 1:**
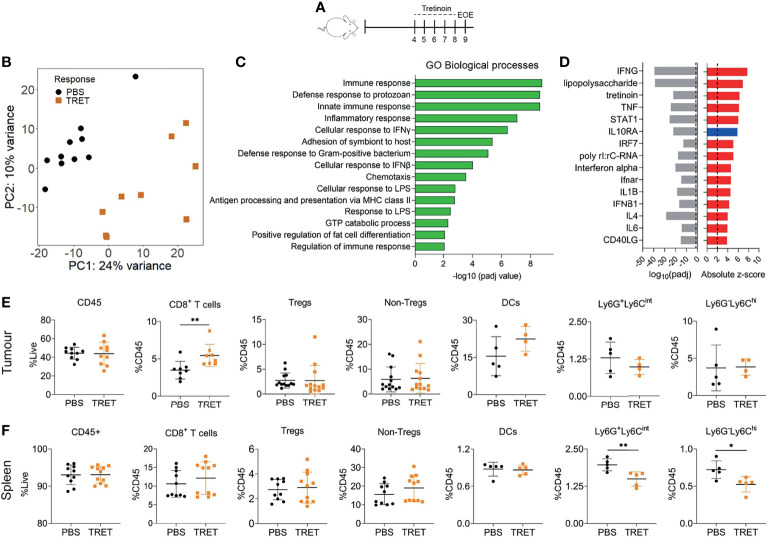
Tretinoin induces an inflammatory, IFN driven tumour microenvironment. **(A)** Experimental design. Mice were inoculated with AB1-HA s.c and treated with 10 mg/kg tretinoin daily for 5 days starting at day 4 when tumours were ~4 mm^2^. Tumours and spleens from tretinoin treated and control (PBS treated) mice were collected for analysis. **(B)** Principal component analysis of tumour derived gene expression data from tretinoin treated (orange square, n=9) or control (black circle, n=10) mice. **(C)** Gene ontology analysis displaying biological pathways upregulated by tretinoin treatment, corrected for multiple comparisons. **(D)** Ingenuity upstream regulator analysis showing absolute z score of positive (red) or negative (blue) upstream regulators of a tretinoin associated gene signature and associated p values. **(E, F)** Proportion of immune cell populations (n=8-13 for lymphoid subsets and n=5 for myeloid subsets) in the tumour **(E)** or spleen **(F)** as determined by flow cytometry. For CIBERSORT and flow cytometry experiments, significance determined using a Mann-Whitney U test corrected for multiple comparisons. *p<0.05 **p<0.01.

We utilised the AB1-HA murine mesothelioma model for this study (31). Subcutaneous AB1-HA tumours display an aggressive, fast growing sarcomatoid pathology with scattered immune infiltration (**Supplementary Figure 2**). Mice were inoculated with AB1-HA mesothelioma cells and treated with 10 mg/kg tretinoin systemically for 5 consecutive days, after which the tumour was harvested (**Figure 1A**). Tretinoin induced a distinct gene expression profile (**Figure 1B**) with 642 genes upregulated and 140 genes downregulated in treated tumours compared to untreated controls (**Supplemental Table 2**). Pathway analysis using Innate DB (26) showed that biological processes associated with the immune response, inflammation, antigen presentation and interferon gamma (IFNγ) signalling were upregulated by tretinoin treatment (**Figure 1C**, **Supplemental Table 3**). We next performed Ingenuity upstream regulator analysis (24) to identify the molecular inducers of this inflammatory, tretinoin-associated gene expression profile. As expected, immune stimulators such as LPS, IFNγ, tumor necrosis factor alpha (TNFα) and type I interferons (IFNs) are upstream regulators predicted to induce this gene expression profile, suggesting that tretinoin has a similar immune stimulating effect (**Figure 1D**).

To determine if there were any specific immune populations associated with this gene expression signature, we performed flow cytometry on tumours and spleens from tretinoin or PBS treated mice. There was no increase in CD45^+^ cells in the tumour (p=0.97) nor spleen (p>0.99) induced by tretinoin (**Figures 1E, F**). Within the tumour, tretinoin increased the proportion of CD8^+^ T cells (p=0.0047 **Figure 1E**). There were no changes to the proportion of other lymphoid nor any myeloid populations (**Supplementary Figure 3**). In the spleen, there was no effect on lymphoid populations (**Figure 1F**, **Supplementary Figure 3**). However, in accordance with other studies (15, 29), tretinoin decreased the proportion of both Ly6G^+^Ly6C^int^ (p=0.0079) and Ly6G^- ^Ly6C^hi^ (p=0.016), CD11b^+^ cells, which have been associated with a MDSC phenotype (32) (**Figure 1F**). We also utilized the RNAseq deconvolution algorithm CIBERSORT, which infers immune cell populations from bulk sequencing data to compare with the flow cytometry data. There was no significant change in the frequency of immune cell populations induced by tretinoin apart from monocytes, which significantly decreased (**Supplementary Figure 4**). Thus, our results show that tretinoin induced inflammation in the tumour microenvironment, increased the ratio of CD8^+^ T cells of CD45^+^ cells and reduced MDSC-like cells systemically, but not in the tumour microenvironment.

### Tretinoin/ICT Combination Efficacy Is Schedule-Dependent

Many clinical and preclinical studies have shown that a pre-treatment inflammatory tumour microenvironment is associated with response to ICT, specifically if driven by type I and II IFNs (9). We therefore hypothesized that tretinoin would sensitize tumours to ICT, testing this by sequencing the treatments, starting with the tretinoin, while adding in the ICT later. We therefore treated AB1-HA tumour-bearing mice with tretinoin and αCTLA-4/αPD-L1 therapy (ICT) concomitantly or in a staggered fashion.

**Figure 2:**
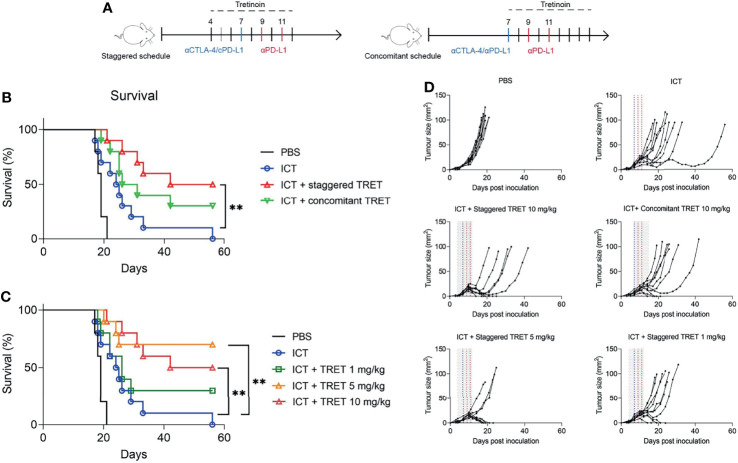
Tretinoin enhances ICT efficacy in a schedule and dose-dependent manner. **(A)** Experimental design. AB1-HA bearing mice were treated with αCTLA-4 on day 7 and αPD-L1 on day 7, 9 and 11. Tretinoin treatment commenced three days prior to (staggered tretinoin, tumour size ~4 mm^2^) or on the same day as ICT (concomitant tretinoin, tumour size ~10 mm^2^) and administered at 10 mg/kg for 9 consecutive days. **(B)** Survival of mice treated with ICT and either staggered or concomitant tretinoin. **(C)** Survival of mice treated with 10 mg/kg, 5 mg/kg or 1 mg/kg tretinoin using the staggered schedule. **(D)** Individual tumour growth of mice treated with tretinoin (grey lines), αCTLA-4 (blue line) and αPD-L1 (red lines). The Log-rank test was used for survival analysis **p<0.01.

We found that tretinoin was most effective at increasing the αCTLA-4/αPD-L1 mediated antitumour response when given in a staggered schedule, with tretinoin treatment commencing 3 days prior to ICT (**Figure 2A**). Survival was significantly increased in mice whose tretinoin treatment begun before ICT compared to ICT alone (**Figure 2B**, p=0.0031). When tretinoin was administered as a concurrent treatment, the combination did not significantly improve survival over checkpoint blockade alone (p=0.118).

Pre-treatment tretinoin was also more effective than concomitance tretinoin at improving ICT efficacy in a second model, RENCA renal cell carcinoma (**Supplementary Figure 5**). Tretinoin was administered *via* oral gavage using a pre-treatment schedule, with tretinoin commencing 5 days prior to ICT, or a concomitant schedule, with tretinoin treatment commencing on the same day as ICT. While the addition of concomitant tretinoin to ICT did not improve survival over either monotherapy (**Supplementary Figure 3**, p= 0.86 compared to ICT and p=0.99 compared to TRET), pre-treatment tretinoin significantly improved survival over tretinoin (p=0.004) or ICT (p=0.006) alone.

Tretinoin has a broad therapeutic window, having been used at a wide range of dosages without significant side effects while having direct anti-cancer effects in acute promyelocytic leukaemia. In order to establish the dose-response relationship for its immunotherapeutic effect, we treated AB1-HA mesothelioma-bearing BALB/c mice with tretinoin at dosages of 1 mg/kg, 5 mg/kg and 10 mg/kg using the staggered schedule (**Figure 2C**). The combination of TRET and ICT at all doses was well tolerated, with no clinical signs of toxicity or weight loss. While 1 mg/kg tretinoin in combination with ICT demonstrated some efficacy with 3/10 responders, this did not result in significantly improved survival (**Figure 2C, D**, p=0.19). However, at the two higher doses, survival was significantly improved (p=0.002 and p=0.003 for 5 mg/kg and 10 mg/kg, respectively). Importantly, the addition of tretinoin to ICT resulted in complete responses (5/10 and 7/10 respectively) in a context where ICT is not curative. Comparing pharmacokinetic studies between humans (33) and mice (34), tretinoin dosages of 5 to 10 mg/kg in mice likely result in biological exposure levels comparable to those observed in humans dosed at the standard dose of 45 mg/m^2^.Together, these results show that tretinoin sensitizes tumours to ICT at clinically relevant dosages.

### Tretinoin Further Enhances the Inflammatory Tumour Microenvironment Induced by ICT

As we observed a strong additive effect of tretinoin and ICT combination therapy, curing 70% of mice in a setting where neither monotherapy is curative, we investigated how the combination treatment changed the tumor microenvironment over either therapy alone.

**Figure 3:**
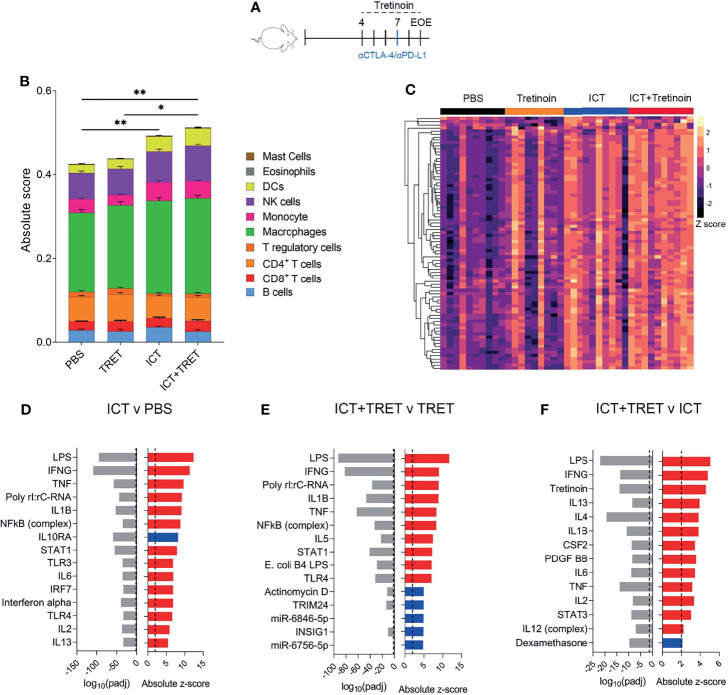
The addition of tretinoin to ICT increases the expression of inflammatory genes and enhances immune infiltration compared to respective monotherapies. **(A)** Experimental design. Mice bearing AB1-HA tumours were treated with PBS, TRET, ICT or TRET+ICT. TRET treatment commenced day 4 (tumour size ~4 mm ^2^) for a total of 4 doses (5 mg/kg) and ICT commenced day 7 (anti-CTLA-4+anti-PDL1) with samples collected on day 9 and RNAseq performed. **(B)** Absolute immune score of tumours as determined by CIBERSORT. **(C)** Heatmap of interferon stimulated genes. Counts were VST normalized and Z scaled. **(D)** Positive (red) or negative (blue) upstream regulators of differentially expressed genes between ICT treated versus control tumours and associated p value (grey). **(E)** Upstream regulators of the differentially expressed genes between TRET+ICT treated tumors versus TRET monotherapy. **(F)** Upstream regulators of differentially expressed genes between TRET+ICT treated tumours and ICT monotherapy treated tumours. PBS (n=10), TRET (n=9), ICT (n=10) or TRET+ICT (n=10). Significance determined using a Mann-Whitney U test corrected for multiple comparisons. *p < 0.05 **p < 0.01.

We performed RNAseq on tumours treated with PBS, tretinoin, ICT or the dual combination (**Figure 3A**). To determine whether the increased efficacy was associated with a change in the immune cell composition of the tumour, we used CIBERSORT (27) to infer immune cell frequencies from the sequencing data. Although we again observed an increase in CD8>^+^ T cells following tretinoin treatment, this didn’t reach significance, which is likely due to a lower resolution of CIBERSORT analysis compared to flow cytometry (35) (**Supplementary Figure 4**). ICT treatment significantly increased the absolute immune score compared to untreated tumours, indicating enhanced immune cell infiltration (**Figure 3B** p=0.003). Although the combination treatment increased immune infiltration compared to control tumours (p=0.003) this change was not significantly different to ICT alone, indicating the effect on immune cell infiltration was largely driven by the ICT component of the combination (p=0.28). While the combination of tretinoin and ICT increased the relative and absolute frequency of most immune populations when compared to untreated or tretinoin treated tumours, this effect was again largely driven by ICT as there was no significant difference between ICT monotherapy and the dual combination (**Supplementary Figure 4**).

Having found only discrete differences in cellular infiltrates for the tretinoin/ICT combination over ICT alone, we queried whether biological pathways were differentially active between the treatment arms. As inflammation and interferon related genes are known to be crucial for a robust response to ICT (9, 36) and we found them to be induced by tretinoin (**Figure 1**), we assessed the expression of interferon stimulated genes across the treatment groups. Untreated control tumours had low expression of interferon stimulated genes (**Supplementary Table 5**), as expected, with tretinoin treated inducing a small increase in expression (**Figure 3C**). ICT treated tumours had high expression of these genes, which was further augmented by the addition of tretinoin, with most tumors expressing high levels of IFNγ genes.

Using Ingenuity upstream regulator analysis, we found that pathways associated with type I and type II interferon signalling were associated with an ICT treated tumour, consistent with previous reports (36, 37) (**Figure 3D**). Similarly, the addition of ICT to tretinoin upregulated genes associated with Toll-like receptor signalling and type I and II interferon activation (**Figure 3E**). The addition of tretinoin to ICT further increased the expression of genes activated by IFNγ, TNFα and other cytokines associated with inflammation (**Figure 3E**), confirming that the combination treatment increased inflammation over ICT alone.

### Activation of Inflammation Distinguishes Responders From Non-Responders Following Tretinoin/ICT Combination Therapy

**Figure 4:**
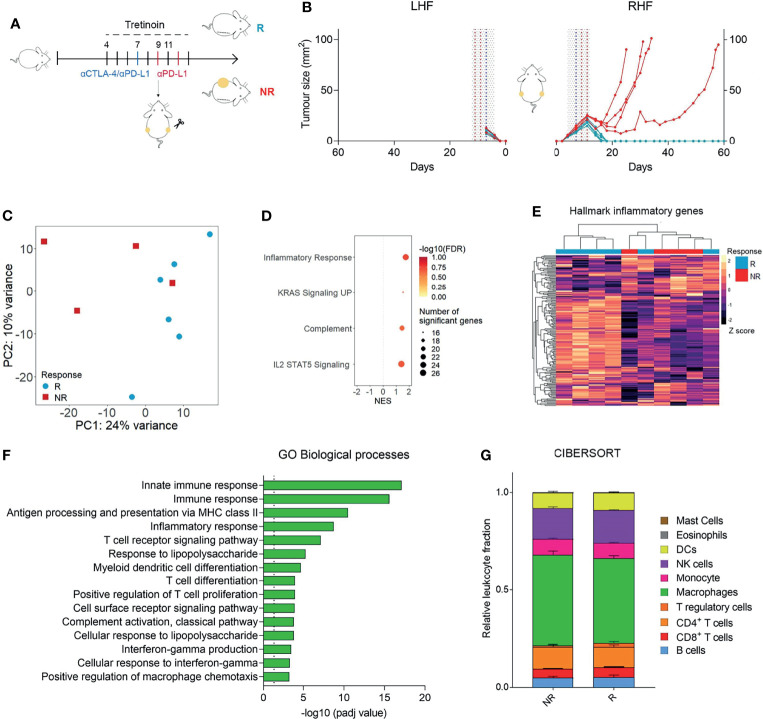
Responding tumours to ICT/ATRA treatment are enriched for inflammatory, IFNy related genes. **(A)** Experimental design. Mice were inoculated bilaterally with AB1-HA and treated with tretinoin (5 mg/kg) followed by αCTLA-4 and αPD-L1 as indicated (tumour size start of treatment ~4 mm^2^). One tumour was surgically resected on day 9 and analysed using RNAseq. The remaining tumour was monitored for treatment outcome; responders (R, blue n=6) or non-responders (NR, red, n=4). **(B)** Tumour growth curves of the unresected right hind flank (RHF) or resected left hind flank (LHF). **(C)** PCA plot of R and NR to tretinoin/ICT. Counts were VST normalized and batch corrected. **(D)** GO biological processes upregulated in responders.**(E)** GSEA analysis of hallmark gene sets upregulated (positive NES) or down regulated (negative NES) in R compared to NR. **(F)** Heatmap displaying the expression of hallmark inflammatory genes, VST normalized and Z scored. **(G)** Relative fractions of cell populations as determined by CIBERSORT.

While the addition of tretinoin to combination CTLA4/PD1 blockade increased the response rate and survival in mice, a proportion (30-40%) of mice still did not respond. To investigate the differences between responders (R) and non-responders (NR) to combination tretinoin/ICT therapy, we utilized the bilateral tumour model (8, 9). Mice were bilaterally inoculated with AB1HA and treated with the staggered tretinoin and ICT schedule (**Figure 4A**). One tumour was then surgically resected five days after the start of tretinoin, which was two days after ICT treatment commenced, with the other tumour remaining *in situ* as an indicator of response (**Figure 4B**). RNAseq was then performed on the excised tumour. Principle component analysis showed a slight separation of responders from non-responders, indicating a difference in the transcriptomic profile between the groups (**Figure 4C**) and differential gene expression analysis identified 379 genes upregulated and 55 genes downregulated in responders compared to nonresponders. Using pathway analysis (**Figure 4D**, **Supplemental Table 4**) and gene set enrichment analysis (**Figure 4E**), we found that the tumours of responders were enriched for inflammatory and immunological pathways and unsupervised clustering on the Hallmarks inflammatory gene set separated most responders from non-responders (**Figure 4F**). Lastly, we investigated whether this inflammatory immune-related expression profile seen in responders was associated with a specific immune cell population. CIBERSORT RNAseq deconvolution showed no difference in the proportions of myeloid or lymphoid immune cells between responders and non-responders (**Figure 4G**) indicating that phenotype of these cells rather than the immune composition of the tumour is associated with treatment outcome, although it is possible that at later time points differences in cellular content will occur.

These data show that tretinoin/ICT combination therapy induces higher levels of inflammatory and immune related pathways in responding mice compared to non-responders. This increased expression of key genes and pathways may be sufficient to drive elimination of the tumour in responders while the lower expression is insufficient induce a robust anti-tumour immune response in non-responders.

### Tretinoin Improves the Anti-Tumour Response to Anti-GITR and Anti-OX40 ICT

**Figure 5:**
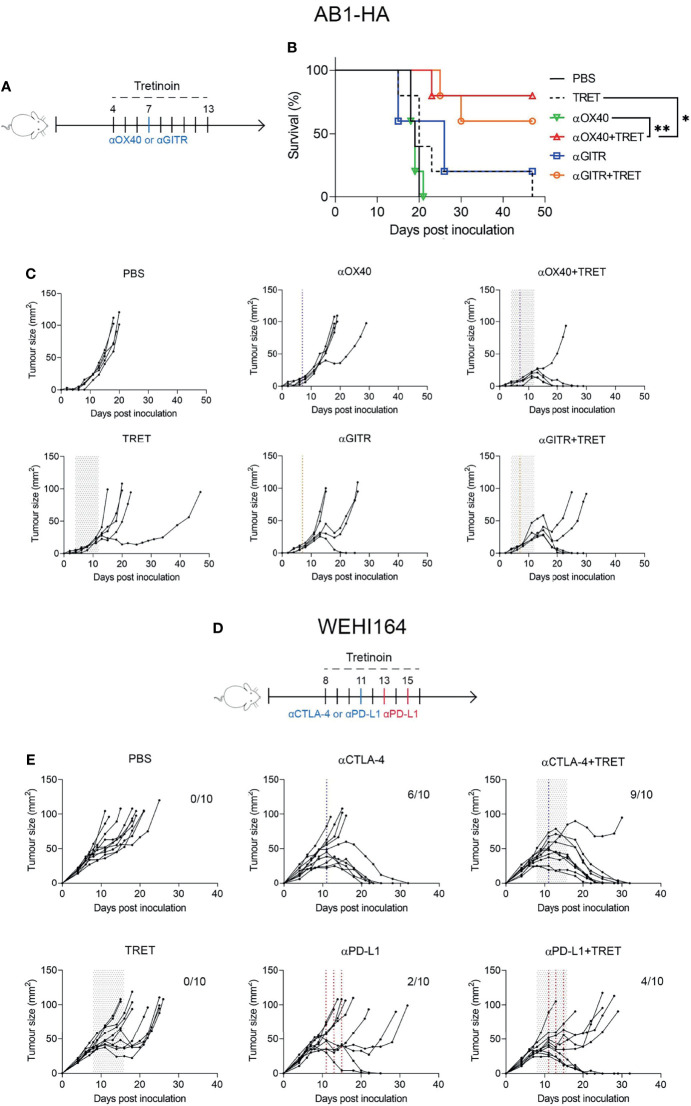
Tretinoin improves the anti-tumour efficacy of single agent ICT in AB1-HA mesothelioma and WEHI164 fibrosarcoma. **(A)** Experimental design. AB1-HA bearing mice were treated with anti-GITR (αGITR) or anti-CD40 (αOX-40) antibodies (day 7), with or without tretinoin dosed in the staggered schedule beginning on day 4 when tumours were ~4 mm^2^ (n=5) . **(B)** Individual tumour growth curves. **(C)** Survival of AB1-HA bearing mice. **(D)** Experimental design. Mice bearing WEHI-164 were treated with CTLA4 (αCTLA-4) or anti-PD-L1 (αPD-L1) on day 11, with or without staggered tretinoin commencing on day 8 when tumours were ~40 mm^2^ (n=10). **(E)** Individual tumour growth curves of WEHI164 inoculated mice treated with ICT and TRET. The log-rank test was used for survival analysis and a mixed model ANOVA for tumour growth analysis. *p < 0.05 **p < 0.01.

Though anti-CTLA-4 and anti-PD-1/PD-L1 are the most clinically utilized immune checkpoint antibodies, there has been robust investigation into the effectiveness of other immune checkpoint targeting antibodies to stimulate an anti-tumour immune response. Agonistic checkpoint antibodies anti-GITR and anti-OX40 have demonstrated strong efficacy as standalone mediators of an anti-tumour response *in vivo* (38) and are in clinical development (39, 40). We next aimed to establish whether tretinoin increased the efficacy of anti-GITR and anti-OX40 therapy. AB1-HA-bearing mice were treated with anti-GITR or anti-OX40 alone or in combination with tretinoin. Neither monotherapy induced a significant anti-tumour immune response (**Figure 5**). However, there was a strong benefit of the addition of tretinoin to anti-OX40, which produced 4/5 complete responses compared to 0/5 for anti-OX40 alone (**Figures 5B, C**). The dual combination of anti-OX40 and tretinoin significantly improved survival (, p=0.0019 against anti-OX40 and p=0.0090 against tretinoin), with delayed tumour growth compared to either treatment alone (, p<0.001 for both comparisons). While the addition of tretinoin to anti-GITR did not enhance survival, there was a significant delay in tumour growth (p<0.001 against tretinoin, p=0.017 against αGITR using mixed model ANOVA) indicating some benefit of the combination treatment.

### Tretinoin Improves ICT Efficacy in the WEHI-164 Fibrosarcoma Model

Lastly, we investigated whether we could validate the efficacy of combining tretinoin with ICT in a second model, and with anti-PD-L1 or anti-CTLA-4 monotherapy, rather than the combination, since ICT is often given as monotherapy in many indications, particularly in the case of antibodies against PD1/PD-L1. For this reason, we chose the WEHI-164 fibrosarcoma model, which displays intermediate sensitive to monotherapy ICT. BALB/c mice were inoculated subcutaneously with WEHI-164 and treated with anti-PD-L1 or anti-CTLA-4 with or without tretinoin administered using the staggered schedule. Interestingly, the addition of tretinoin changed the response profiles, increasing response rate from 6/10 to 10/10 and increasing the cure rate from 6/10 to 9/10 (**Figures 5D, E**). Similarly, although to a lesser extent, this was seen for anti-PD1, increasing response rate from 2/10 to 5/10 and complete cures from 2/10 to 4/10 (**Figure 5E**). Mixed model ANOVA analysis showed that the addition of tretinoin to αCTLA4 significantly delayed tumour growth compared both anti-CTLA-4 monotherapy (p=0.014) and tretinoin monotherapy (p<0.001).

## Discussion

Together, our data demonstrate that in addition to previously reported peripheral depletion of immunosuppressive MDSCs (14, 15), tretinoin induces an ICT responsive tumour microenvironment characterized by inflammation, CD8^+^ T cell infiltration, and decreased accumulation of intra-tumoural Tregs. Additionally, the level of inflammation induced by the combination treatment is associated with response, as complete responders to the ICT/Tretinoin combination were enriched for inflammatory and IFNy related genes when compared to non-responders.

Alongside its direct anti-tumour capability (41), tretinoin has a range of immunomodulatory effects that can decrease immunosuppression (13). Tretinoin has been reported to induce the differentiation of immunosuppressive MDSCs into non-immunosuppressive macrophages or dendritic cells (14, 15). As high levels of MDSCs are associated with a decreased response to ICT (42, 43), depleting these cells prior to the commencement of immunotherapy could improve response and this strategy has shown promise *in vivo* (29). While we did not see the depletion of MDSCs within the tumour microenvironment, both granulocytic and monocytic MDSCs were depleted in the spleen, consistent with other reports (29, 30). Tretinoin is also a powerful regulator of Treg activity and can induce Foxp3 expression in peripheral CD4^+^ T cells, converting them to Tregs as well as increase the number of tumour infiltrating Tregs (12, 44), although we saw no changes to CD4^+^ Treg and non-Treg populations.

Similar to our findings, it has been previously reported that tretinoin can increase CD8^+^ T cell infiltration into the tumour (11). Tretinoin treated CD8+ T cells also have increased expression of effector molecules, IFNy, granzyme B, perforin and increased presence of cell migration markers (11, 12). As higher levels of CD8^+^ T cells are associated with a better response to ICT (31, 45), the positive effects of tretinoin on CD8^+^ T cell infiltration could be exploited to enhance ICT efficacy. While the effects of tretinoin on immune cell populations are well known, the effects of tretinoin on the transcriptomic profile of a whole tumour has been less studied. We performed RNAseq to characterize the transcriptomic changes induced by tretinoin and found that tretinoin treated tumours were enriched for inflammatory and IFNy related genes and pathways. This is in line with another study that found tretinoin upregulated genes associated with IFN response and antigen presentation (46).

Increased T cell infiltration, inflammation and decreased MDSC frequencies are characteristics of an immunogenic ‘hot’ tumour microenvironment (47). Inflammatory ‘hot’ tumours are more responsive to ICT (36, 48) and this phenotype could be used as a clinical biomarker for ICT response. However not every tumour has an inflammatory, ICT sensitive phenotype, with as low as 5% and as high as 50% of patients having a pre-existing inflammatory gene signature, depending on cancer type (49, 50). Therefore, it is necessary to investigate ways to therapeutically induce this gene expression profile which in turn could increase the number of a patients that respond to ICT. The validity of this strategy has been demonstrated *in vivo*, with the intratumoural administration of vaccines or recombinant cytokines able to improve ICT efficacy through the induction of an inflammatory phenotype (9, 51). The clinical translation of these treatment protocols are limited, as intratumoural injections are logistically difficult in many cancer types. Our data demonstrate that tretinoin is a useful alternative that can achieve the same effect.

Since it can be administered orally and is already used widely in the clinic, it does not have the disadvantages of intratumoural therapies and it can be translated swiftly into the clinic. Two other *in vivo* studies also demonstrated that the addition of tretinoin to anti-PD-1 improved the anti-tumour immune response in murine models of colon cancer, breast cancer and lung cancer, further confirming the robustness of the results (29, 30). A small clinical trial in melanoma patients investigated treatment of tretinoin at high dose during 4 days surrounding the dosing of either anti-CTLA4 or anti-PD1, which showed excellent tolerability (52). We are the first to show that tretinoin can increase the efficacy of antibodies against the emerging immune checkpoints OX40 and GITR, both of which are in early stage clinical trials (39, 40).

We acknowledge some limitations to our study. To achieve a level of background responsiveness to ICT in the murine models similar to that in patients, treatment had to begin early after inoculation when the tumour size was relatively small. This early induction of therapy is different to the situation in patients, where tumours have developed *in situ* for an extended period. Additionally, tumor size can affect treatment efficacy (53) and although tretinoin had minimal effect on its own, we cannot fully exclude some size-dependent effect between our staggered and concomitant schedule, where treatment (with tretinoin) commenced three days earlier.

Finally, while the combination of tretinoin and ICT was very efficacious, still a proportion (~30%) of mice did not respond to treatment. We utilized the bilateral tumour model (8, 9) to characterize the on-treatment differences in the tumour microenvironment between responders and non-responders. Complete responders to tretinoin/ICT had significantly higher levels of inflammation compared to non-responders. As inflammation is a key contributor to ICT efficacy (48, 54), this suggests that the responders reached a threshold of inflammation which enhanced the anti-tumour immune response enough to induce complete regression, while this was insufficient in non-responders. The magnitude of expression induced by treatment could be utilized as an on-treatment biomarker of response to aid in clinical decision making.

In conclusion, we show that as well as depleting peripheral MDSCs, tretinoin induces an inflammatory, IFN rich, ‘hot’ tumour microenvironment that is sensitive to ICT. Tretinoin pretreatment improves the efficacy of both single and dual agent ICT in both murine mesothelioma and fibrosarcoma. Additionally, the level of inflammation induced by the tretinoin/ICT combination correlates with response, further substantiating the necessity of inflammation for a robust anti-tumour immune response induced by ICT. The clinical availability and tolerability of tretinoin suggests this drug is a good potential candidate to enhance responses to checkpoint blockade.

## Data Availability Statement

The original contributions presented in the study are publicly available. This data can be found here: https://www.ncbi.nlm.nih.gov/geo/, GSE186195.

## Ethics Statement

The animal study was reviewed and approved by Harry Perkins Institute for Medical Research animal ethics committee.

## Author Contributions

CT, TC and EJ contributed equally to this paper. CT, TC, RZ, JS and GW performed mouse experiments. CT, EJ, AB analyzed RNA sequencing data. CT analyzed flow cytometry data. AB, MM, AN, RL, and WL interpreted experiments and critically revised the manuscript. CT, TC and WL wrote a first draft of the paper. RL and WL designed the study, supervised the project and edited the manuscript. All authors contributed to the article and approved the submitted version.

## Funding

This work was supported by Douglas Pharmaceuticals. WL was supported by fellowships from the Simon Lee Foundation, NHMRC (grant names APP1126076 and APP1196605) and Cancer Council WA. The National Centre for Asbestos Related Diseases received funding through the NHMRC Centre of Research Excellence scheme (grant number APP1197652).

## Conflict of Interest

EJ, AB, RZ, RL and WL are co-inventors on patents relating to aspects of this manuscript (WO2016015095A1 and WO2019178650A1). AB, MM, AN, RL and WL have received consultancy fees from Douglas Pharmaceuticals.

The remaining authors declare that the research was conducted in the absence of any commercial or financial relationships that could be construed as a potential conflict of interest.

## Publisher’s Note

All claims expressed in this article are solely those of the authors and do not necessarily represent those of their affiliated organizations, or those of the publisher, the editors and the reviewers. Any product that may be evaluated in this article, or claim that may be made by its manufacturer, is not guaranteed or endorsed by the publisher.
