# Supplementary material for: Retinoic Acid Induces an IFN-Driven Inflammatory Tumour Microenvironment, Sensitizing to Immune Checkpoint Therapy
Source: Front Oncol. 2022 Mar 24;12:849793. doi: 10.3389/fonc.2022.849793 (PMC8988133; doi:10.3389/fonc.2022.849793)
Supplement: Supplementary file 2 [file DataSheet_2.pdf]

SUPPLEMENTARY FIGURES

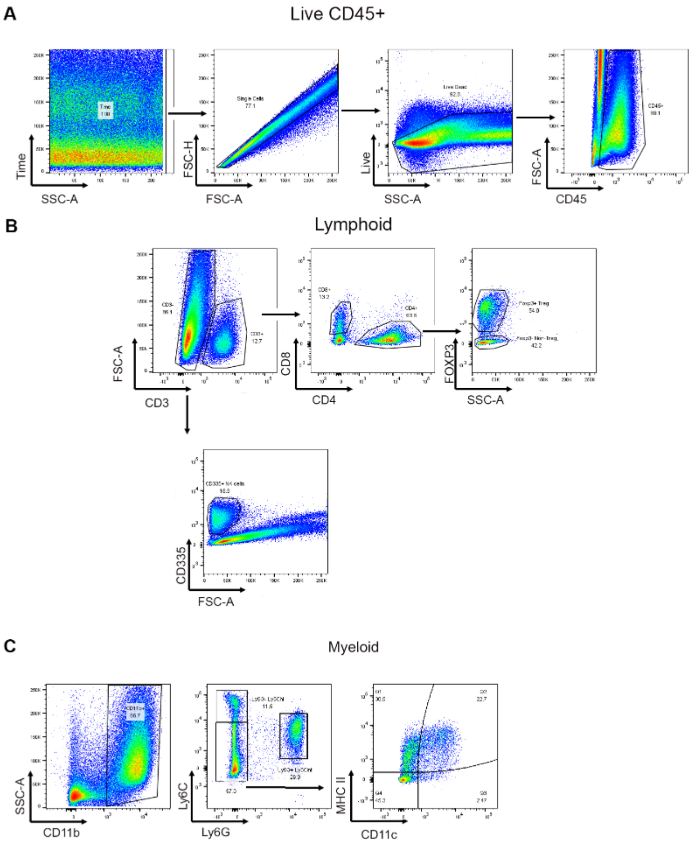

Supplementary figure 1. Example flow cytometry gating strategy. (a) Live CD45$^+$ cells were first selected. Lymphoid (b) or myeloid (c) populations were then gated. Both tumor and spleen samples were gated following this strategy with the above figure showing a representative spleen sample.

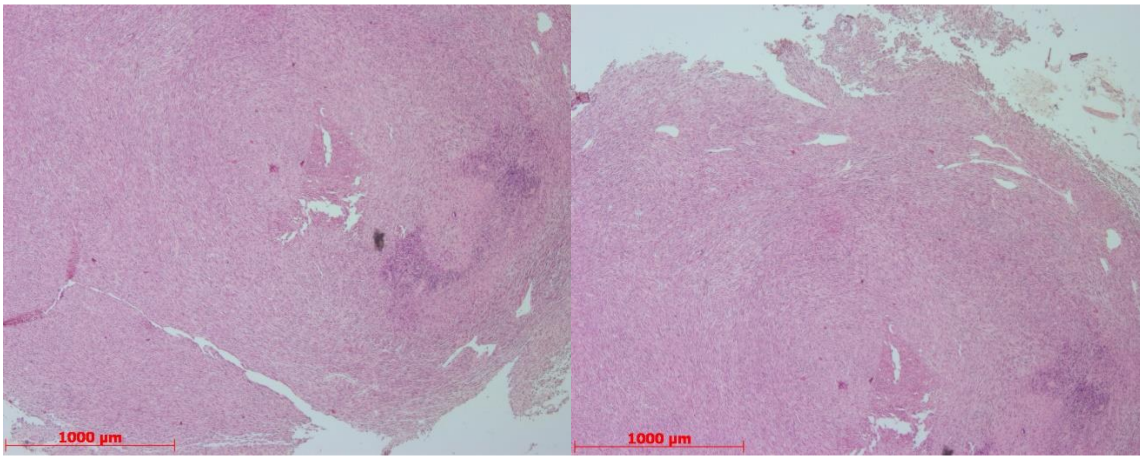

Supplementary figure 2. H&E stained sections of subcutaneous AB1-HA murine mesothelioma tumors. BALB/c mice were inoculated with AB1-HA s.c and tumors excised 9 days post inoculation and embedded in paraffin for histologic analysis. Samples were stained using hematoxylin and eosin.

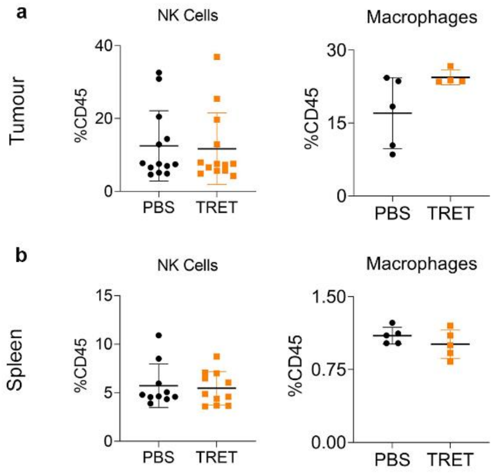

Supplementary figure 3. Extended flow cytometry data. Tumour (a) or Spleen (b) cells were gated as in Supplementary figure 1.

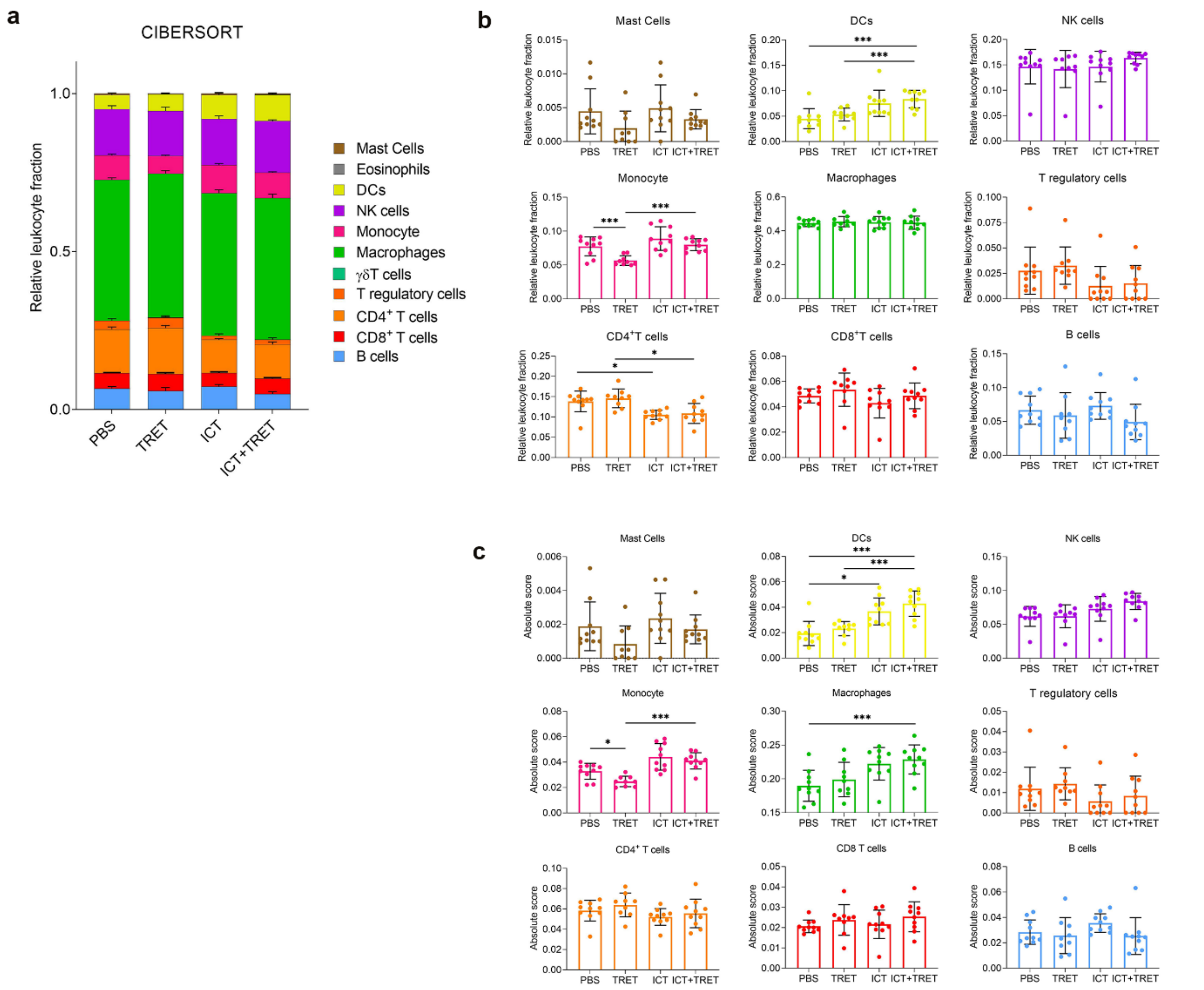

Supplementary figure 4. Extended CIBERSORT data. Immune cell populations as determined using CIBERSORT of AB1-HA tumours. (a-b) Relative proportions of immune populations. (c) Absolute proportions of immune cell populations. Significance determined using a Mann-Whitney U test corrected for multiple comparisons. *p<0.05 **p<0.01 ***p<0.001

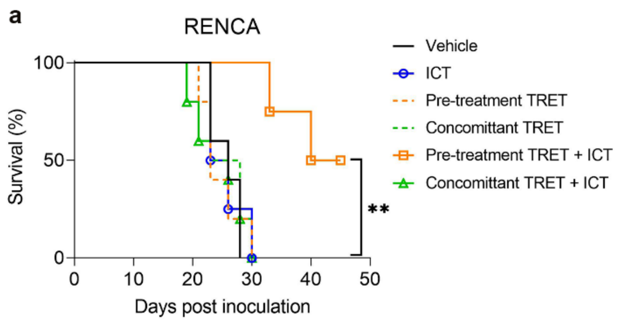

Supplementary figure 5. Tretinoin pre-treatment sensitizes RENCA to ICT. (a) Survival curve of RENCA bearing mice treated with αCTLA-4 on day 12 and αPD-L1 on day 12, 14 and 16 i.p. Tretinoin was dosed as a pre-treatment regime commencing five days prior to immune checkpoint therapy (ICT) or as a concomitant regime commencing on the same day as ICT and administered at 10 mg/kg in DMSO and soybean oil via oral gavage. The Log-rank test was used for survival analysis **$p<0.01$.

Supplementary table 1. Lymphoid and myeloid flow cytometry panels.

		Myeloid		
**Marker**	**Fluorochrome**	**Clone**	**Company**	**Catalogu**
**CD45**	BUV395	30F11	BD	564279
**MHC II**	AF700	M51.114.15.2	BioLegend	107622
**CD11b**	BV711	M1/170	BioLegend	101241
**CD11c**	PE-CY7	N418	eBioscience	25-0114-82
**LY6C**	BV421	HK1.4	BioLegend	128032
**LY6G**	FITC	IA8	BioLegend	127606
**Zombie UV**	Viability	1:1000	-	Biolegend
		Lymphoid		
**CD45**	FITC	30F11	BD	553080
**CD3**	BUV395	145-2C11	BD	563565
**CD4**	BV786	GK1.5	BD	563331
**CD8**	PE-CY7	53-6.7	BD	553877
**CD25**	BV421	PC61	BioLegend	102043
**CD335**	BV711	29A1.4	BioLegend	137621
**FOXP3**	AF700	MF-14	BioLegend	126422
**ICOS**	PE	7E.17G9	eBioscience	12-9942-82
**Ki67**	BV605	16A8	BioLegend	137621
**ZombieUV**	Viability	1:1000	-	Biolegend

Supplementary table 2. Differentially expressed gene analysis using DESeq2. Genes were considered differentially expressed with an adjusted p value of <0.05 and absolute log fold change ≥1.

**Comparison**	**Number of differentially expressed genes**
**Tretinoin vs PBS**	642 upregulated by tretinoin, 140 downregulated by tretinoin
**ICT vs PBS**	916 upregulated by ICT, 115 downregulated by ICT
**Tretinoin+ICT vs ICT**	272 upregulated by Tretinoin+ICT,83 downregulated by Tretinoin+ICT

Supplementary table 3. GO biological pathways enriched in tretinoin treated tumours compared to PBS (control) tumours and the DEG associated with these pathways.

**Pathway Name**	**Gene Symbols**
Immune response	Ccl17; Ccl22; Ccl24; Ccl6; Ccr1; Ccr4; Cd24a; Csf2; Cxcl2; Enpp2; Gbp6; H2-Ab1; H2-Bl; H2-DMa; H2-DMb2; H2-Oa; H2-Q10; Il1a; Il5; Irf8; Prg2; Smad3; Tgtp1; Tgtp2; Tinagl1; Tnfrsf1b; Tnfsf10; Tnfsf13b;
Defense response to protozoan	Batf2; Gbp2; Gbp3; Gbp5; Gbp6; Gbp7; Gbp9; Irf4; Irf8; Irgm2; Slc11a1;
Innate immune response	Atf3; C1ra; C1rb; C5ar1; Ccl17; Cd200; Cd300lf; Cd40; Cebpb; Clec7a; Clec9a; Csf2; Cxcl2; Dusp10; Fcna; Gbp2; Gbp6; Gbp7; Gm5077; Gpr77; H2-Ab1; Hspa1b; Ido1; Igtp; Ikbke; Il1a; Il5; Irak2; Irf1; Irf5; Irf8; Irgm1; Lair1; Ms4a8a; Nlrc5; Nod1; Nrip1; Ntn1; P2rx7; Padi4; Slc11a1; Tbx21; Tgtp1; Ticam2; Tlr12; Tnfaip3; Tnfrsf9; Treml4; Trim21; Trpm2; Unc93b1; Vegfa; Zbp1;
Inflammatory response	Bmp2; C5ar1; Ccl22; Ccl24; Ccr1; Ccr4; Ccrl2; Cd40; Chi3l3; Chi3l4; Clec7a; Cxcl2; Gpr77; Ido1; Il1a; Il5; Kcnj10; Ncf1; P2rx7; Ptafr; Slc11a1; Themis2; Ticam2; Tlr12; Tnfaip3; Tnfrsf1b;
Cellular response to interferon-gamma	Gbp2; Gbp3; Gbp4; Gbp5; Gbp6; Gbp7; Gbp8; Gbp9; H2-Ab1; Il12rb1;
Adhesion of symbiont to host	Gbp2; Gbp3; Gbp5; Gbp6; Gbp7; Gbp9;
Defense response to Gram-positive bacterium	C5ar1; Gbp2; Gbp3; Gbp5; Gbp6; Gbp7; Gbp9; Ncf1; Nod1; P2rx7; Tnfrsf14;
Cellular response to interferon-beta	Gbp2; Gbp3; Gbp5; Gbp6; Gm4951; Igtp; Irf1;
Chemotaxis	Ccl17; Ccl24; Ccr4; Ccrl2; Cmklr1; Cmtm8; Cxcr7; Ear2; Enpp2; Gpr77; Ptafr;
Cellular response to lipopolysaccharide	Cd40; Csf2; Cx3cr1; Cxcl2; Gbp2; Gbp6; Gfi1; Irf8; Ticam2; Tnfaip3; Tnfrsf1b;
Antigen processing and presentation of exogenous peptide antigen via MHC class II	H2-Ab1; H2-DMa; H2-DMb2; H2-Oa; Unc93b1;
Response to lipopolysaccharide	Adm; Alpl; C5ar1; Ccr1; Cebpb; Dusp10; Ido1; Il10ra; P2rx7; Ptafr; Ptger2; Slc11a1; Tnfrsf1b; Vcam1;
GTP catabolic process	Gbp11; Gbp2; Gbp3; Gbp4; Gbp5; Gbp6; Gbp7; Gbp8; Gbp9; Gimap7; Igtp; Irgm2; Rab15; Rab20; Rab37; Rasl11a; Tgtp1; Tgtp2;
Positive regulation of fat cell differentiation	6330406I15Rik; Bmp2; Cebpb; Cmklr1; Frzb; Lrp5;
Regulation of immune response	Card11; Cd40; Smad3; Tbx21; Tnfsf13b;

Supplementary table 4. Enriched GO Biological pathways in the tumours of responders compared to non-responders and the DEG associated with these pathways

Pathway Name	Upregulated DEG in pathway
Innate immune response	Anpep; Atf3; Bnip3; C1ra; C1rb; C1rl; C3; Ccl17; Ccl5; Cd300lf; Cd40; Cd86; Cebpb; Clec7a; Clec9a; Coro2a; Csf2; Ctss; Cxcl9; Glrx; Gm5077; H2-Aa; H2-Ab1; Hspa1b; Icam1; Ido1; Il13; Il1a; Il27; Irf8; Itgax; Itgb2; Klrg1; Lair1; Malt1; Nfil3; Nos2; Pglyrp1; Pik3cd; Ppargc1b; Prkcd; Rarres2; Samhd1; Serpinb2; Serping1; Siglecg; Stat4; Tbkbp1; Tbx21; Ticam1; Ticam2; Tlr12; Tnfrsf9; Trpm2;
Immune response	Ccl11; Ccl17; Ccl22; Ccl24; Ccl5; Ccl8; Ccr1; Ccr7; Cd24a; Cd274; Cd28; Cd4; Csf2; Cxcl9; Enpp2; Fcgr2b; Gm7030; H2-Aa; H2-Ab1; H2-DMa; H2-DMb1; H2-DMb2; H2-Eb1; H2-Oa; Il13; Il1a; Irf8; Lta; Ltb; Zap70;
Antigen processing and presentation of exogenous peptide antigen via MHC class II	Ctse; Fcgr2b; H2-Aa; H2-Ab1; H2-DMa; H2-DMb1; H2-DMb2; H2-Eb1; H2-Oa;
Inflammatory response	C3; Ccl22; Ccl24; Ccl5; Ccl8; Ccr1; Ccr7; Cd40; Clec7a; Cnr2; Cxcl9; Ido1; Il1a; Il27; Lta; Nos2; Pik3cd; Pla2g7; Rarres2; Themis2; Ticam1; Ticam2; Tlr12;
T cell receptor signaling pathway	Bcl2a1d; Cd28; Clec2i; Itk; Malt1; Skap1; Themis; Themis2; Txk; Zap70;
Response to lipopolysaccharide	Ccr1; Ccr7; Cd86; Cebpb; Cnr2; Icam1; Ido1; Il13; Il18bp; Lta; Mapkapk3; Nos2; Ptger2; Ptgir; Ticam1;
Myeloid dendritic cell differentiation	Batf2; Batf3; Cd86; Csf2; Irf4; Ubd;
T cell differentiation	Bcl2a1d; Cd3d; Cd4; Chd7; Gimap1; Rhoh; Tbx21;
Positive regulation of T cell proliferation	Card11; Ccl5; Ccr7; Cd28; Cd86; Il2ra; Itgal; Pdcd1lg2;
Cell surface receptor signaling pathway	Cd22; Cd24a; Cd274; Cd3d; Cd4; Fcgr2b; Fcgr3; Gpr97; Itgal; Klrg1; P2rx5; Pik3cd;
Complement activation, classical pathway	C1ra; C1rb; C1rl; C3; Gm5077; Serping1;
Cellular response to lipopolysaccharide	Cd40; Cd86; Csf2; Cyp11a1; Gfi1; Icam1; Irf8; Nos2; Ticam2; Tnip3;
Interferon-gamma production	Eomes; Itk; Runx3; Txk;
Cellular response to interferon-gamma	Ccl5; Gbp5; Gbp8; H2-Ab1; Il12rb1; Nos2;
Positive regulation of macrophage chemotaxis	Ccl5; Ccr7; Cmklr1; Rarres2;

Supplementary table 5. Interferon stimulated gene set used in RNAseq analysis.

**Interferon stimulated genes**			
Sod2	Sppl2a	Upp1	Isg15
Cd274	Fcgr1	Psmb10	Oas2
Cfb	Sp140	Gbp6	Xaf1
Cd40	Psme1	Gbp10	Oas1g
Myd88	Tap1	Gbp4	Ddx58
Samhd1	Stat1	Ccl2	Ddx60
Trim30d	Psmb8	Wars	Usp18
Ripk1	B2m	Rtp4	Bst2
Il6	Psmb9	Gbp2b	Oas1b
Socs1	Samd9l	Gbp2	Mx2
Mvp	Nmi	Gbp9	Pnpt1
Parp14	Trim30a	Trim21	Ifit3
Irf7	Oasl1	Casp1	Eif2ak2
Tapbp	Gzma	Ogfr	Tdrd7
Znfx1	Ido1	Ifitm3	Ifit3b
Cxcl10	Cxcl11	Ifi44	
Batf2	Dhx58	Apol6	
Ptgs2	Irf9	Parp12	
Trim5	Pml	Stat2	
Trim12c	Gbp11	Cmpk2	
Uba7	Rsad2	Isg20	
Casp7	Oas3	Adar	
C1rb	Parp9	Ifih1	
Sectm1a	Zbp1	Mov10	
Tmem140	Psme2	Oas1a	
